# Supplementary material for: Probabilistic thermodynamic analysis of metabolic networks
Source: Bioinformatics. 2021 Mar 23;37(18):2938–45. doi: 10.1093/bioinformatics/btab194 (PMC8479673; doi:10.1093/bioinformatics/btab194)
Supplement: btab194_supplementary_data [file btab194_supplementary_data.pdf]

Supplementary Information

Probabilistic Thermodynamic Analysis of Metabolic Networks

Mattia G. Gollub, Hans-Michael Kaltenbach, Jörg Stelling

1 Methods

1.1 Probabilistic Metabolic Optimization (PMO)

We solved PMO problems using the Gurobi Optimizer v9.0.1 (Gurobi Optimization, Beaverton, OR). Eq. (1) summarizes the constraints:

$$\begin{aligned} \mathbf{t} := \begin{bmatrix} \ln \mathbf{c} \\ \mathbf{\Delta_r G'^\circ} \\ \mathbf{\Delta_r G'} \end{bmatrix} &= \mathbf{Q}\mathbf{m} + \mu_\mathbf{t} \\ \|\mathbf{m}\| &\leq \chi^2_n(\alpha) \\ \mathbf{S} \cdot \mathbf{v} &= 0 \\ \mathbf{lb} \leq \mathbf{v} &\leq \mathbf{ub} \\ M_f \mathbf{d} - \mathbf{v_{con}} &\leq \mathbf{1} \cdot (M_f - \epsilon_f) \\ M_f \mathbf{d} - \mathbf{v_{con}} &\geq \mathbf{1} \cdot \epsilon_f \\ M_r \mathbf{d} + \mathbf{\Delta_r G'} &\leq \mathbf{1} \cdot (M_r - \epsilon_r) \\ M_r \mathbf{d} + \mathbf{\Delta_r G'} &\geq \mathbf{1} \cdot \epsilon_r \end{aligned} \tag{1}$$

Here, the vector of integers $\mathbf{d}$ in $\{0,1\}^\gamma$ denotes if a reaction has positive (1) or negative (0) flux and the relationship between free energies and fluxes has been rewritten in the *Big-M* formulation [20]. M_f (M_r) are the big-M values and ϵ_f (ϵ_r) are the minimum magnitudes for fluxes (reaction energies). These values must be chosen carefully as *Big-M* constraints are often a source of numerical errors. In most cases, the range of coefficients should not span more than 9 orders of magnitude. We choose $M_r = 1000 \frac{kJ}{mol}$ as it is larger than the $\Delta_r G'^\circ$ of any reaction in the model and $\epsilon_r = 0.1 \frac{kJ}{mol}$ since smaller values imply high enzyme cost and are thus unlikely. For fluxes, we set M_f larger than the highest flux magnitude (1000 in iML1515-CAN) and $\epsilon_f = 10^{-8} \cdot M_f$. Additionally we set the Gurobi parameters `FeasibiltyTol` $= 10^{-9}$ and `IntFeasTol` $= 10^{-9}$ to ensure that sign constraints are not violated.

The objective depends on the application: for quantitative assessment of the models we minimize $\|\mathbf{m}\|_2$ (i.e. we maximize the probability of $\mathbf{m}$), while for searching initial points for the sampler we maximize and minimize reaction fluxes. We used $\alpha = 0.95$ as confidence level.

The matrix $\mathbf{Q}$ and the vector of z-scores $\mathbf{z}$ of a solution $\mathbf{m}$ are computed from the truncated eigenvalue decomposition of $\mathbf{\Sigma_t}$

$$\mathbf{\Sigma_t} = \mathbf{U}\mathbf{\Lambda}\mathbf{U}^\intercal \approx \mathbf{U_q}\mathbf{\Lambda_q}\mathbf{U_q}^\intercal = \mathbf{U_q}\mathbf{\Lambda_q}^{1/2} \cdot \left(\mathbf{U_q}\mathbf{\Lambda_q}^{1/2}\right)^\intercal , \tag{2}$$

where $\mathbf{U}$ is an orthogonal matrix containing the eigenvectors of $\mathbf{\Sigma_t}$ and $\mathbf{\Lambda}$ is a diagonal matrix containing the eigenvalues of $\mathbf{\Sigma_t}$. $\mathbf{U_q}$ and $\mathbf{\Lambda_q}$ are submatrices of $\mathbf{U}$ and $\mathbf{\Lambda}$ that represent eigenvectors and eigenvalues for which the eigenvalue is larger than 10^{-3}. Given the decomposition, $\mathbf{Q} = \mathbf{U_q}\mathbf{\Lambda_q}^{1/2}$ and $\mathbf{z} = \mathbf{U_q}\mathbf{m}$.

1.2 Thermodynamics and Flux Sampling (TFS)

The algorithm for sampling $\mathcal{T}$ (see main text) relies on the ability to quickly verify whether an orthant in thermodynamic space is *feasible*, i.e. there is a steady state flux solution that satisfies the directionality constraint of the orthant. We achieve fast feasibility verification with the following optimizations:

1. First, we test a simple condition that is necessary but not sufficient at steady state: for each metabolite, there must be at least one in-flux and one out-flux. This is the case when the system of equations

$$|\mathbf{S} \cdot sign(\mathbf{v})| < \sum_j |\mathbf{S_j}| , \tag{3}$$

where $\mathbf{S_j}$ are the columns of $\mathbf{S}$, is satisfied.

2. We maintain a fixed-size cache (10^5 entries) with Least Recently Used (LRU) policy that stores the results of the linear program. If a set of directions has been already tested and the result is present in the cache, we use the result directly without calling the solver.

3. If Eq. (3) and the cache cannot determine the feasibility of an orthant, we verify feasibility with a linear program

$$\begin{aligned} \max_{\mathbf{v}} \quad & \mathbf{0} \\ \text{s.t.} \quad & \mathbf{S} \cdot \mathbf{v} = 0 \\ & \mathbf{lb_{orth}} \leq \mathbf{v} \leq \mathbf{ub_{orth}} \ , \end{aligned} \tag{4}$$

where the $\cdot_{orth}$ subscript indicates the original flux bounds restricted to the directions of the orthant. The optimization problem can be solved quickly because (1) setting the objective to zero, the solver only needs to find one feasible point instead of the optimum and (2) we reuse the same model object for each orthant and only modify the flux bounds. This way the solver can use information from the previous solution.

For each simulation on iML1515-CAN we run 200 chains (starting from different modes of the thermodynamic space) for $2 \cdot 10^8$ steps. The first half of the random walk is treated as warm-up and discarded. From the remaining steps, we collect at regular intervals a total of 10^5 samples of $\mathbf{\Delta_r G'}$ and 10^8 direction samples. We then use the samples of $\mathbf{\Delta_r G'}$ to conditionally sample metabolite concentrations and $\mathbf{\Delta_r G'^{\circ}}$. However, due to memory requirements it is more efficient to characterize the probability of each orthant using the signs of the reversible reactions only. These are stored efficiently in a hashmap were the keys are binary serializations of the sign pattern of the reversible reactions and the values indicate the number of times an orthant was sampled.

1.2.1 Validation

We implemented two samplers for TFS: A non-convex, non-uniform sampler for $\mathcal{T}$ and a convex uniform sampler based on Coordinate Hit and Run with Rounding (CHRR) for sampling orthants of $\mathcal{F}$. The samplers are implemented in C++, and elementary operations (ray-polytope intersections, ray-ellipsoid intersection, sampling from 1D uniform distribution, sampling from 1D truncated normal distribution) are covered by unit tests. To verify that the implementations sample from the correct distributions we performed a set of validations against established approaches:

1. **Uniform sampling**: we uniformly sampled the *e_coli_core* model and iML1515-CAN (growth on glucose) using our implementation and the COBRA Toolbox implementation. We used the same number of chains, samples, warmup steps and total steps for both implementations. Figure SI 2 shows that the two implementations have similar convergence properties and predict the same distributions.

2. **Multivariate Normal (MVN) sampling**: we sampled MVN distributions of different dimensions with random means and covariances using our implementation and the built-in MATLAB function `mvnrnd`. Figure SI 3 shows that samples generated with our sampler are as good as samples generated using `mvnrnd`.

3. **Sampling of $\mathcal{T}$**: we sampled a toy network using TFS and a simple rejection-based approach. In the latter, we generated random samples of free energies using `mvnrnd` and then used a simple linear program (based on the constraints in Eq. (1), where the integer variables are already fixed by the reaction energies) to reject samples that did not satisfy the steady state condition. Figure SI 4 shows that, for the toy network, both implementations generate comparable sets of samples. In this example with only five internal reactions, the rejection rate was 88.2%. We can assume that each additional irreversible reaction reduces the steady-state thermodynamic space by half, thus doubling the number of rejections, and the example shows that reversible reactions increase the number of rejections as well. Thus, rejection sampling quickly becomes prohibitive even for core models and cannot be used to evaluate results obtained with TFS on iML1515-CAN. However, the comparison on the toy network combined with the asymptotic guarantees of hit-and-run [2] and the practical convergence results (Section SI 3.1) suggest that TFS samples the thermodynamic space correctly even for large models such as iML1515-CAN.

4. **Sampling the flux space in TFS**: since sampling of the flux space is achieved by uniformly sampling multiple orthants independently, this step is validated through point (1).

1.3 Models

The condition-specific models used in the analysis (iML1515-CAN) were generated with the following steps:

1. We used *NetworkReducer*'s lossy reduction [5] allowing removal of the reactions, metabolites in the following subsystems: *Cell Envelope Biosynthesis*, *Glycerophospholipid Metabolism*, *Lipopolysaccharide Biosynthesis / Recycling*, *Membrane Lipid Metabolism*, *Cofactor and Prosthetic Group Biosynthesis*, *Folate Metabolism*, *Murein Biosynthesis*, *Murein Recycling*. Additionally, we allowed for removal of transport and exchange reactions for

metabolites that are present exclusively in the subsystems above. During reduction, we protected the model's capability of reproducing the measured growth and exchange rates. Reducing the level of detail in these subsystems was necessary to make the model computationally tractable and to ignore parts of the network where the thermodynamic model could be unreliable (e.g. different phase and poorly defined metabolites in lipid metabolism).

2. We manually removed/lumped reactions that only participate to secretion of metabolites or energy-wasting loops in the subsystems above.

3. We removed reactions related to oligosaccharides (glycogen and maltose) as their production and degradation form unfeasible cycles at steady state and the annotated directionalities are thermodynamically inaccurate.

4. PFK_3 and FBA3 were removed to make fluxes in the pentose phosphate pathway comparable to the 13C estimates.

5. We integrated experimental data (metabolite concentrations when required and measured growth/exchange rates).

6. The model was further reduced using *NetworkReducer* lossless compression. We protected all reactions in carbon, amino acid and nucleotide metabolism, which we believed were the most important in the growth conditions (minimal media).

7. As we require non-zero flux through all reactions modeled with thermodynamic constraints, we removed all blocked reactions.

The resulting models maintain an intact description of carbon, amino acid and nucleotide metabolism. Intracellular metabolomics data were only used for model assessment (glucose and acetate conditions) and in the $M+$ conditions. In all conditions we set extracellular concentrations according to the composition of M9 media.

2 Thermodynamic assessment of iML1515-CAN

Table SI 1 lists the irreversible reactions that we had to make reversible to avoid thermodynamic inconsistencies. The same inconsistencies were found in all growth conditions. Table SI 2 shows the anomalies found for the growth on glucose and acetate, together with the explanation we found based on literature and the curation steps. We curated the model only in presence of support from literature. It is possible that some of the irreversibility annotations that we removed during the curation process were added to the model because it is known that *E. coli* does not actively use these reactions in the opposite direction. This is an intentional choice, as thermodynamic reversibility and regulatory choices are two different kinds of constraints. While knowledge of the preferred direction of a reaction can be added on top of a thermodynamically constrained model (e.g. for further reducing the number of modes predicted by TFS), this should not be used as a thermodynamic constraint.

Solving the PMO optimization problem took a time varying between 1 and 4 minutes on a Intel® i7-8700K processor depending on the growth condition and whether the results of the curation were applied or not.

3 Sampling iML1515-CAN

3.1 Convergence and performance

For all six conditions, the random walks satisfied recommended convergence metrics [6] (split-R ≤ 1.1 and $ESS < 5 \cdot n_c$, where n_c is the number of chains). Note that we computed these statistics on the split-chains, meaning that, after discarding the warm-up steps, we split each chain in two halves and treated each half as a chain. This is recommended to detect systematic trends in the chains. After sampling the thermodynamic space, we randomly selected 10^4 modes according to their probabilities and used our implementation of CHRR to sample their flux spaces. The number of flux samples drawn for each orthant was proportional to its probability.

Table SI 3 shows a comparison of the runtimes of Uniform Sampling (US) and TFS sampling a core model and iML1515-CAN. Benchmarks were executed on our HPC cluster consisting of 26 nodes, each equipped with two Intel® Xeon® E5-2697 processors (year 2013, 12 cores at 2.70 GHz) and 128 GB of memory. On this system we used 8 cores when sampling the core model and 200 cores when sampling iML1515-CAN. We chose to simulate 200 chains (one per core) for iML1515-CAN to have higher chances of detecting issues in their convergence. However, this is not a general requirement and fewer chains can be simulated on smaller systems, as long as there is sufficient confidence that the sampler is not missing large portions of the space (e.g. for models as large or smaller than iML1515-CAN and with a similar fraction of reversible reactions). TFS simulations take significantly longer than US, increasing the runtime from a few minutes to almost a day for iML1515-CAN. Most of the runtime of TFS was spent simulating hit-and-run, a task that unfortunately can not be parallelized for individual chains. However, sampling a core model required less

than 5 minutes, suggesting that sampling models containing few hundred reactions is computationally affordable on simple desktop computers. Moreover, the runtime depends mostly on the dimensionality of the thermodynamics space and less on the dimensionality of the flux space. If an application requires investigation of specific areas of the network only, one can apply thermodynamic constraints only to the corresponding reactions, resulting in faster simulations.

3.2 Prediction of directions and flux distributions

Table SI 4 shows the number of orthants in each condition. These cover a wide range of behaviors, which reflect on the flux distributions of individual reactions (Figure SI 5, Figure SI 6). Interestingly, US overlooks many of the capabilities of the network. This is likely an artifact of representing the flux space with a polytope. In high dimensions, most of the volume lies in the center of the space and orthants that only appear close to the boundary of the polytope are too unlikely to be found, independent of their thermodynamic probability.

Table SI 5 summarizes the results of the validation of precision and accuracy of US and TFS against 13C estimates. However, 13C estimates have limited coverage and there were several cases where US and TFS predicted the irreversibility of a reaction but in different directions. We manually validated the predicted directions using EcoCyc [11] (Table 6 and 7) and found that in all verifiable cases TFS predicts the correct direction.

3.3 Prediction of metabolite concentrations

For comparisons against TMFA we used the matTFA [16] implementation, modified to use $\mathbf{\Delta_r G'^\circ}$ estimates from eQuilibrator. We constrained the estimates of metabolite concentrations and $\mathbf{\Delta_r G'^\circ}$ to their 95% confidence interval. Figure SI 7 shows the predicted TFS distributions and Thermodynamics-based Metabolic Flux Analysis (TMFA) ranges for each metabolite with measured concentration. TMFA could not constrain the concentration of any of those metabolites. This is consistent with the results obtained by the authors of TMFA, which showed constrained metabolite ranges only assuming no error in the standard reaction energies [9].

4 Supplementary figures and tables

Table SI 1: List of the irreversible reactions in iML1515-CAN that we had to make reversible to avoid thermodynamic inconsistencies.

Reaction	Reason
ACCOAL	Enforces unfeasible internal cycle.
ACt4pp	Conflicts with ACt2rpp and NAt3pp.
GLYCLTt4pp	Conflicts with GLYCLTt2rpp and NAt3pp.
PPAt4pp	Prevents excretion of propionate, enforcing an unfeasible internal cycle.
PPCSCT	Enforces unfeasible internal cycle.
PROt4pp	Conflicts with PROt2rpp and NAt3pp.
PTA2	Enforces unfeasible internal cycle.

Table SI 2: Interpretation of the PMO results. NA denotes values that are not available because the metabolite was removed after model curation.

Metabolite	z-score		Concentration (mM)		Explanation
	Before	After	Before	After	
Metabolites with $\lvert z \rvert \geq 1$ for growth on glucose.					
pi_c<br>pi_e	−2.8<br>1.4	−0.1<br>0.0	14.2<br>64.0	24.6<br>55.7	Import of the default phosphate specie (HPO_4^{2-}) through PIt2rpp is thermodynamically unfeasible at the given concentrations. Phosphate must be transported either as $H_2PO_4^-$ or through PIuabcpp. **Action:** Make PIt2rpp reversible and add reaction for the transport of $H_2PO_4^-$ [12].
mqn8_c<br>mql8_c	2.4<br>−2.4	0.8<br>−0.8	30.4<br>0.002	1.3<br>0.05	The reactions of the respiratory complex I have been found to be reversible in mitochondria [18]. It is likely that reversibilities apply also in the bacterial orthologs. **Action:** Make the reactions NADH16pp, NADH17pp and NADH18pp reversible, as their irreversibility annotation is thermodynamically inaccurate.
aspsa_c	−2.4	NA	0.002	NA	Substrate channeling has been shown experimentally between ASPK, ASAD and HSDy [10]. We hypothesize channeling through ASPK, ASAD and DHDPS as the respective enzymes are predicted to bind [17]. **Action:** Convert ASPK, ASAD, HSDy and DHDPS to the lumped reactions ASPK_ASAD_HSDy and ASPK_ASAD_DHDPS.

Table SI 2: Interpretation of the PMO results. NA denotes values that are not available because the metabolite was removed after model curation.

Metabolite	z-score		Concentration (mM)		Explanation
	Before	After	Before	After	
5caiz_c<br>5aizc_c	2.0<br>−2.0	NA<br>0.0	14.6<br>0.004	NA<br>0.25	We hypothesize channeling of 5caiz_c between AIRC2 and AIRC3, since the respective enzymes purK and purE have been shown to bind in *E. coli* [17]. Note that this is controversial [15]. **Action:** Convert AIRC2 and AIRC3 to the lumped reaction AIRC2_AIRC3.
o2_c	−1.7	−1.7	0.008	0.008	Expected. Limited by extracellular o2 concentration. **Action:** None.
3pg_c<br>3php_c	1.6<br>−1.6	0.26<br>−0.26	6.4<br>0.010	0.42<br>0.15	PGCD alone is thermodynamically unfavorable. SerA has been shown to couple 3pg dehydrogenation to akg reduction in *Pseudomonas*. The same mechanism is likely employed by *E. coli* as well [22]. **Action:** Make PGCD reversible. Add reactions for the proposed mechanism: 3pg_c + akg_c $\rightleftharpoons$ 3php_c + r2hglut_c and akg_c + q8h2_c $\rightleftharpoons$ r2hglut_c + q8_c.
acg5p_c<br>acglu_c	−1.5<br>1.5	0.0<br>NA	0.012<br>5.2	0.25<br>NA	Channeling of acglu between ACGS and ACGK has been shown experimentally in *S. cerevisiae* [1], we hypothesize a similar mechanism in *E. coli.* **Action:** Convert ACGS and ACGK to the lumped reactions ACGS_ACGK.
glu5p_c	−1.5	NA	0.012	NA	Channeling of glu5p from GLU5K to G5SD is experimentally supported in *E. coli* [14]. **Action:** Convert GLU5K and G5SD to the lumped reaction GLU5K_G5SD.
4abut_c<br>sucsal_c	1.5<br>−1.5	0.0<br>NA	5.1<br>0.013	0.25<br>NA	Physical interaction between the enzymes catalyzing ABTA and SSALx has been shown experimentally in mitochondria [8]. We hypothesize a similar interaction in *E. coli* leading to channeling of sucsal for both SSALx and SSALy. **Action:** Convert ABTA, SSALx and SSALy to the lumped reactions ABTA_SSALx and ABTA_SSALy
ser__L_c<br>2amsa_c	1.5<br>−1.5	0.0<br>NA	4.7<br>0.014	0.25<br>NA	Artifact of the steady-state constraint. 2amsa is a required intermediate for the synthesis of D-serine from L-serine. However, it is unlikely that *E. coli* actively synthesizes D-serine as it is not involved in any cellular function. **Action:** Remove 2amsa_c and ser__D_c to avoid imposing unrealistic constraints on the concentration of L-serine.
succoa_c<br>coa_c<br>sl2a6o_c<br>thdp_c	1.3<br>−1.2<br>−1.1<br>1.1	0.60<br>−0.33<br>0.0<br>NA	3.2<br>0.0245<br>0.027<br>2.4	0.83<br>0.13<br>0.25<br>NA	Inaccuracies in group contribution estimates. DHDPS is a ring-forming reaction synthesizing 23dhdp, which is then converted to thdp. THDPS then opens the thdp ring, forming sl2a6o. Both 23dhdp and thdp are not involved in any reaction in TECRDB, thus their formation energy must be estimated from group contribution, which is known to be unreliable for ring alterations [4]. **Action:** Ignore the ring thermodynamics by replacing DHDPS, DHDPRy and THDPS with the lumped reaction DHDPS_DHDPRy_THDPS.
ptrc_c<br>4abutn_c	1.3<br>−1.2	1.1<br>−1.1	3.1<br>0.021	2.3<br>0.027	STRING predicts possible binding between PatA an PatD. However, we could not find further evidence supporting substrate channeling between the two enzymes. **Action:** None.
gcald_c<br>4hthr_c<br>gly_c	1.1<br>−1.1<br>1.1	0.20<br>NA<br>0.0	2.5<br>0.026<br>2.5	0.39<br>NA<br>0.25	As 4hthr does not participate in any essential pathway and its maximum flux is very low, the validity of the constraints from the directions of 4HTHRA and 4HTHRK is questionable. *in-vivo* those may be affected by transport and dilution. **Action:** Remove 4HTHRA and 4HTHRK to avoid imposing unrealistic constraints on the concentration of glycine.
acon_c	−1.0	−1.0	0.030	0.030	(1) Citrate dehydratase is an unfavourable reaction, while the previous step (citrate synthase) is predicted to be highly favourable ($\Delta_r G' - 40.5 \frac{kJ}{mol}$), suggesting channeling of citrate between the two reactions. Indeed there is experimental evidence for a mitochondrial enzyme complex including malate dehydrogenase, citrate synthase and aconitase [3, 21]. We hypothesize a similar interaction in *E. coli*, leading to the channeling of oxaloacetate and citrate. (2) It is not clear whether aconitate is channeled between the two aconitase steps, since it must be released from the enzyme and turn 180° before binding again. [13] We conservatively assume it is. **Action:** Add lumped reactions for the potential channels (1) MDH_to_CS, CS_to_ACONT, MDH_to_ACONT and (2) ACONTa_ACONTb .
Additional metabolites with concentration $\geq 10 mM$ for growth on glucose.					
cl_p	0.57	0.3	75.5	6.0	The annotated irreversibility of CLt3_2pp is thermodynamically inaccurate and implies high periplasmic concentrations of cl_L. **Action:** Make CLt3_2pp reversible.
glu__L_p	0.46	0.0	26.2	0.25	The annotated irreversibilities of GLUt4pp, GLUABUTt7pp and ABUTt2pp are thermodynamically inaccurate and imply high periplasmic concentrations of glu__L. **Action:** Make GLUt4pp, GLUABUTt7pp and ABUTt2pp reversible.
Additional metabolites with $\lvert z \rvert \geq 1$ for growth on acetate.					

Table SI 2: Interpretation of the PMO results. NA denotes values that are not available because the metabolite was removed after model curation.

Metabolite	z-score		Concentration (mM)		Explanation
	Before	After	Before	After	
glyc_c	2.2	0.36	19.9	0.52	Gluconeogenesis appears unfavourable at the given intracellular concentrations. *in-vivo*, unfavourable reactions could be overcome by substrate channeling (see main text). To make unfavourable reactions feasible, they would need to be coupled with favourable steps (PPCK and FBP). **Action:** Based on evidence from literature and predicted requirements we hypothesised two complexes performing substrate channeling: (1) PPCK, ENO, PGM, PGK, GAPD, TPI (or any subset starting from PPCK) and (2) FBP, FBA, TPI (or any subset starting from FBP). The thermodynamics of glycolysis is highly influenced by magnesium ions [19], currently not accounted for by eQuilibrator. While it is likely that some form of channeling involving FBP and PPCK occurs in the pathway, the exact composition of the enzyme complexes is hard to determine. The predicted complexes are thus possibly larger than the actual ones.
g3p_c	−1.5	−1.7	0.012	0.007	
dha_c	1.4	−0.36	4.2	0.12	
ac_c	1.5	1.3	4.8	3.6	Plausible given the growth condition. **Action:** None.
g1p_c	−1.2	−1.2	0.022	0.023	STRING predicts possible binding between Pgm an Pgi, but we could not find further literature evidence supporting substrate channeling between the two enzymes. Moreover, glucose-6-phosphate isomerase operates close to equilibrium, thus substrate channeling between the two enzymes would not bring any thermodynamic advantage. **Action:** None.
sucgsa_c	−1.0	−0.87	0.032	0.044	STRING predicts possible binding between AstC an AstD, however we could not find further literature evidence supporting substrate channeling between the two enzymes. **Action:** None.
sucorn_c	1.0	0.87	2.0	1.4	

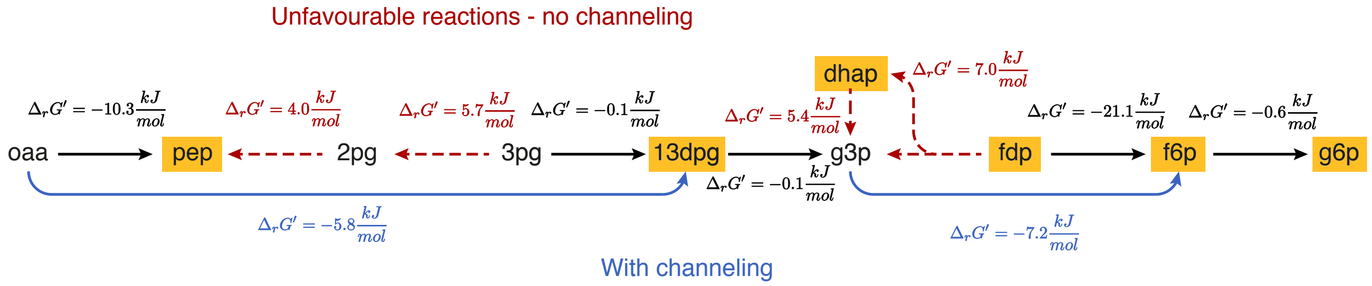

Figure SI 1: Overview of the predicted reaction energies in gluconeogenesis. The concentrations of the metabolites highlighted in orange, as well as all cofactors other than CO2 and phosphate were constrained by measurements. Phosphate is constrained to literature values. Reactions predicted to be unfavorable are shown in red. An example of hypothetical net reactions caused by substrate channeling is shown in blue.

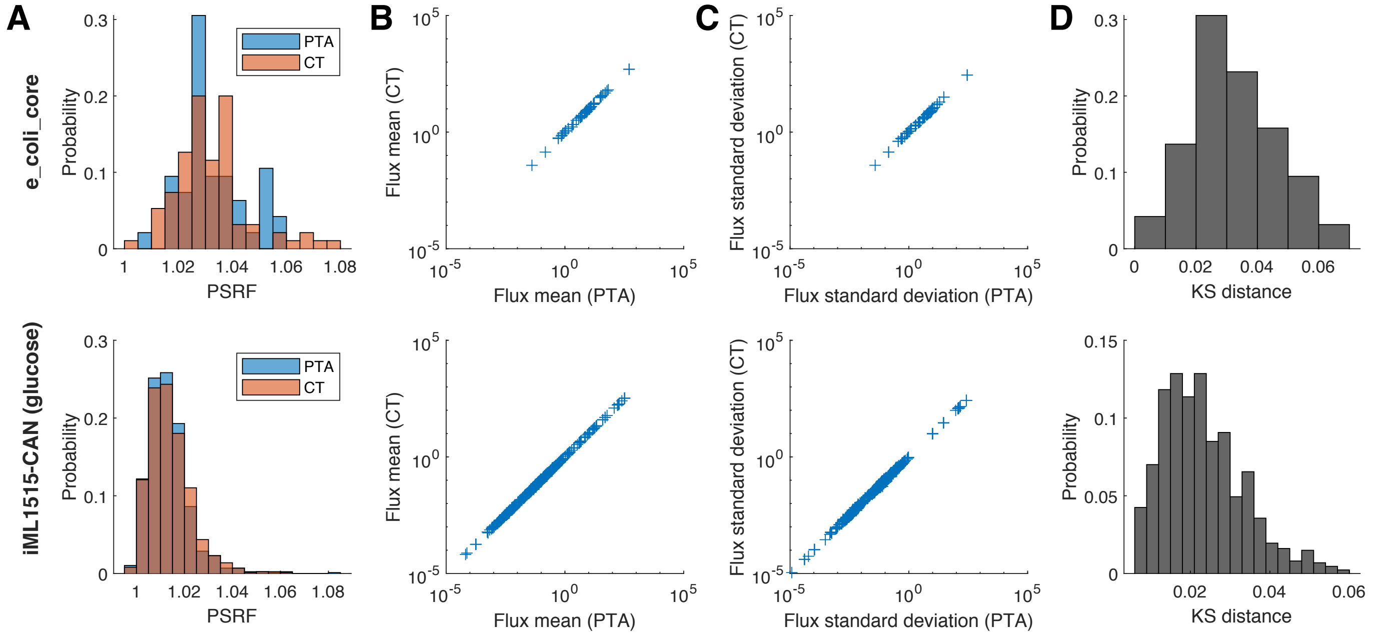

Figure SI 2: Comparison between our implementation of CHRR (PTA) and the implementation in the COBRA Toolbox (CT) for the *e_coli_core* and *iML1515-CAN* (growth on glucose) models. (**A**) Both implementations have similar convergence properties, as shown by the Potential Scale Reduction Factors (PSRFs). (**B**, **C**) Both implementations predict the same mean and standard deviation for the probability distribution of each flux. (**D**) Kolmogorov-Smirnov (KS) distance between the distributions predicted by the two implementations for each flux.

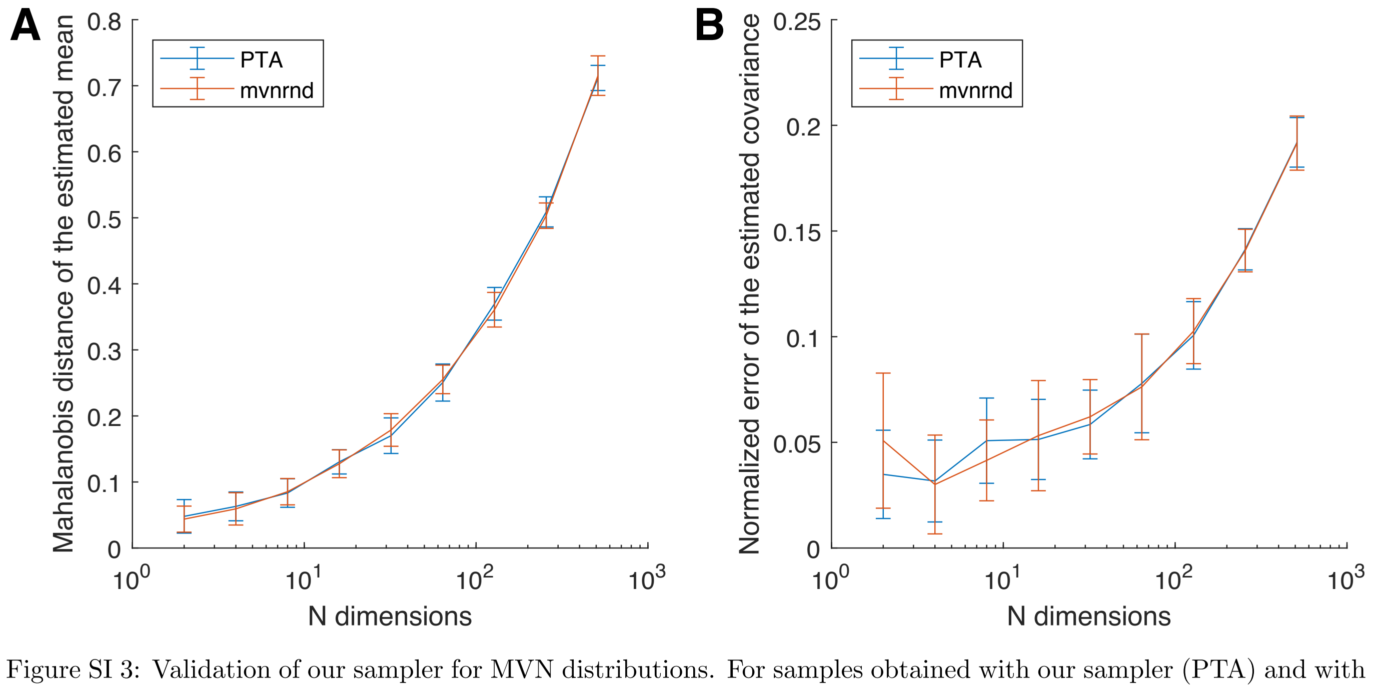

Figure SI 3: Validation of our sampler for MVN distributions. For samples obtained with our sampler (PTA) and with the built-in MATLAB function `mvnrnd` we compare (**A**) the Mahalanobis distance between the true means and the means estimated from samples and (**B**) the Frobenius norm between the true covariance and the covariance estimated from the samples, normalized by the Frobenius norm of the true covariance. Error bars represent the standard deviation over 10 replicates, each with random mean and covariance.

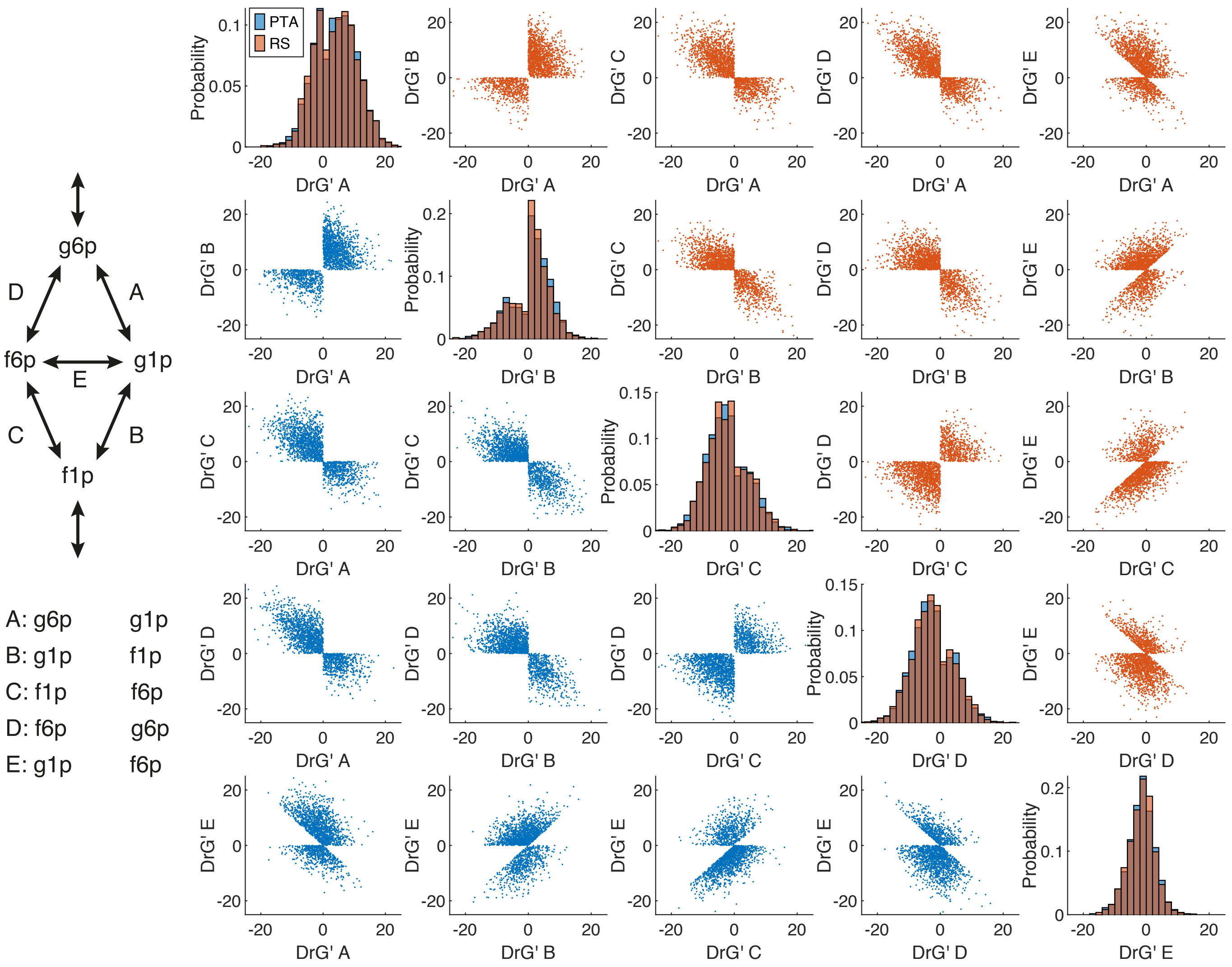

Figure SI 4: Validation of TFS sampling the thermodynamic space of a hypothetical toy network (left). The four metabolites (glucose 6-phosphate (g6p), glucose 1-phosphate (g1p), fructose 6-phosphate (f6p), fructose 1-phosphate (f1p)) have similar formation energies, thus all reaction energies are distributed around zero. However, steady state imposes additional constraints on the reaction energies. For example, if conversion of g6p to f1p through reactions A and B is favourable, then conversion through D and C must be favourable as well. We show the distribution of the samples for each reaction energy (diagonal) and pair of reaction energies (off-diagonal) predicted by our sampler (PTA, blue) and by a simple rejection sampling approach (RS, orange).

Table SI 3: Runtime of TFS (including individual steps of the pipeline) and US. Benchmarks show the average over the six $M+$ conditions for iML1515-CAN and a core model based on *e_coli_core* and modified to include reactions for the measured exchanges [7]. For both models, the number of reactions for TFS and US differ because some results of thermodynamic curation would have allowed additional internal or ATP-generating cycles and were thus not applied for US.

	e_coli_core (modified)	iML1515-CAN
Number of cores	8	200
Number of chains	100	200
Number of reactions (US)	$89 - 92$	$864 - 871$
Number of reactions (TFS)	$101 - 104$	$875 - 883$
**TFS**	4 min 43 s ± 7 s	23 h 46 min ± 54 min
Sampling $\mathbf{\Delta_r G'}$	4 min 22 s ± 4 s	19 h 47 min ± 47 min
• Finding initial points	29 s ± 4 s	4 h 3 min ± 33 s
• Iterative rounding	2 min 35 s ± 5 s	2 h 1 min ± 6 min
• Hit-and-run	49 s ± 1 s	12 h 47 min ± 32 min
Sample $\ln \mathbf{c}$	0 s ± 0 s	22 s ± 1 s
Sampling flux orthants	22 s ± 11 s	3 h 58 min ± 17 min
**US**	3.4 s ± 0.3 s	4 min 54 s ± 30 s

Table SI 4: Number of orthants found in each condition. The last column shows the minimum number of orthants required to cover 95% of the thermodynamic space.

Metabolomics	Condition	N. orthants	Mean samples per orthant	% orthants for 95% coverage
M-	fru	17761983	5.63	71.85%
M-	glyc	13952353	7.17	66.39%
M-	pyr	17058219	5.86	70.69%
M-	succ	17099759	5.85	70.76%
M+	fru	10092355	9.91	62.25%
M+	glyc	8760400	11.42	60.88%
M+	pyr	14382468	6.95	67.01%
M+	succ	12381754	8.08	65.49%
M+	ac	10539012	9.49	63.12%
M+	glc	11596903	8.62	64.58%
M-	Average:	16468079	6.13	70.11%
M+		11292149	9.08	64.21%

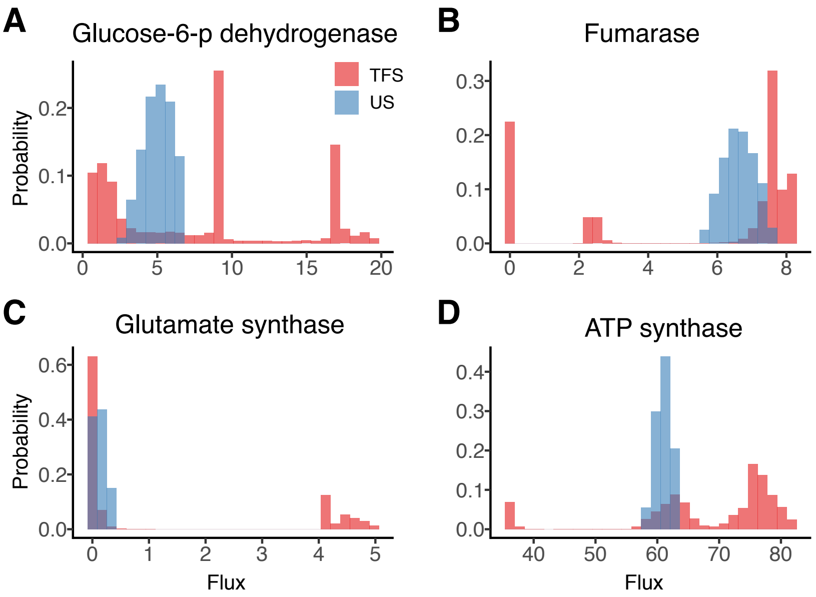

Figure SI 5: Selected examples comparing the flux distributions predicted by US and TFS ($M-$, growth on fructose). (**A**) Large variability in the flux through the pentose phosphate pathway. (**B**) A "branched TCA" (inactive or reverse fumarase) is themodynamically realistic. (**C**) *E. coli* has two paths for synthesizing glutamate. The path through glutamate synthase requires more ATP than the path through glutamate dehydrogenase, but may be the only thermodynamically feasible option in low-nitrogen conditions. (**D**) The flux through oxidative phosphorylation can vary significantly depending on the directions selected in the rest of the network.

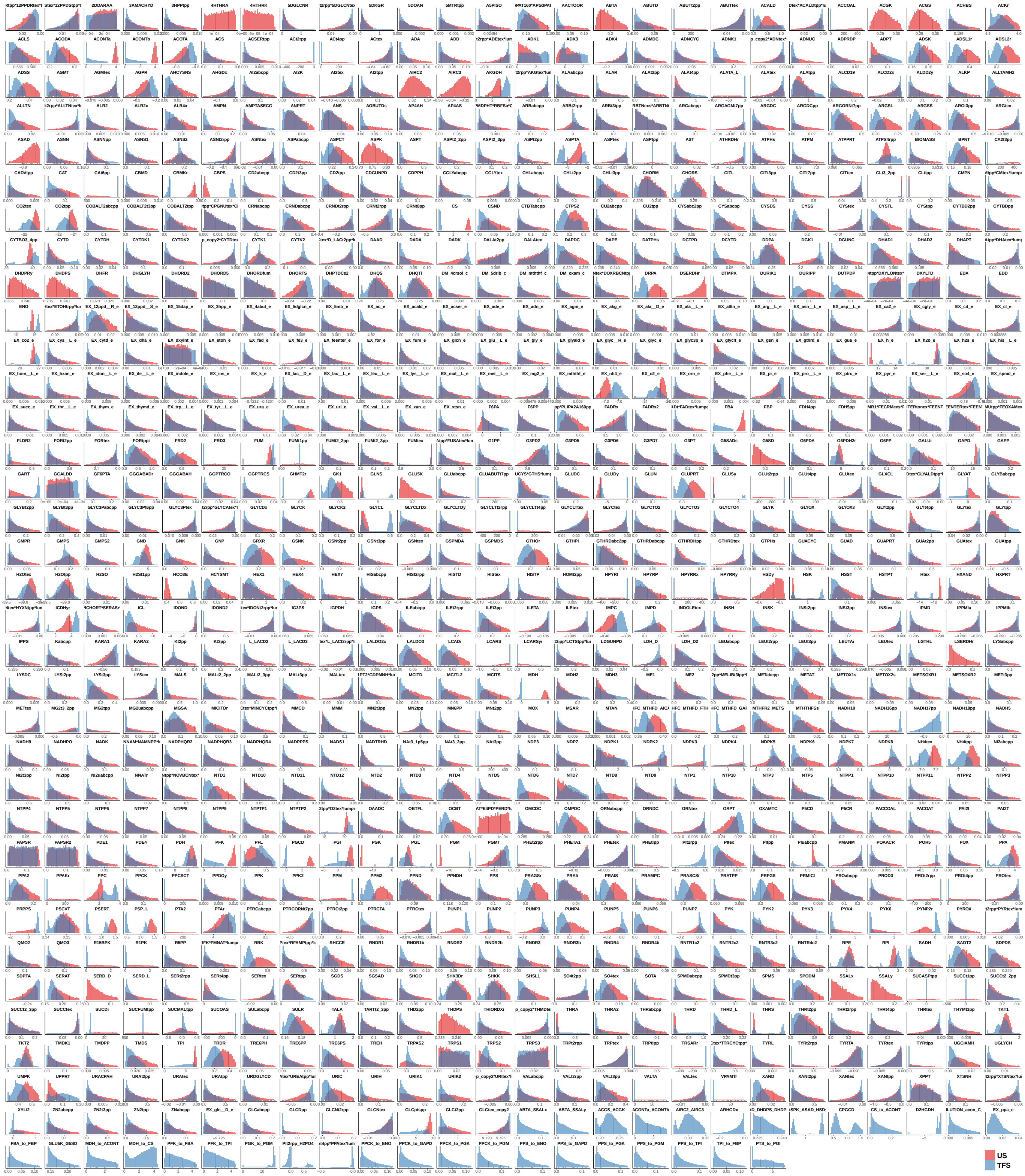

Figure SI 6: Fluxes for all the reactions in iML1515-CAN (growth on glucose, $M+$) predicted with US and TFS.

Table SI 5: Comparison of precision and accuracy of different methods (see main text). Counts (percentages) are given as range (mean) over all conditions. The last two rows report the number (percentage relative to the reversible reactions) of reactions for which US and TFS predicted irreversibility in different directions.

	TMFA		TMFA+US		TFS	
	M-	M+	M-	M+	M-	M+
Rxns in model	864-870	864-871	864-870	864-871	875-881	875-883
Reversible rxns in model	73-79	71-79	73-79	71-79	102-105	102-105
Reversible fluxes in model	11-14	10-14	11-14	10-14	13-15	13-15
Predicted reversible reactions	71-77	57-70	32-40	28-35	53-57	46-48
(% of reversible rxns in model)	97.7%	83.5%	49.2%	42.5%	53.3%	45.5%
Predicted irreversible reactions (% of reversible rxns in model)	2.3%	16.5%	50.8%	57.5%	46.7%	54.5%
Incorrect predicted irreversibilities	0	0-3	0-5	0-3	0	0
(% of reactions covered by 13C estimates)	0.0%	4.1%	14.0%	8.2%	0.0%	0.0%
Conflicting (TMFA + US vs TFS)					11-18	9-13
(% of reversible rxns in US model)					17.2%	14.7%

Table SI 6: Evaluation of the predicted directions of reactions for which US and TFS predicted irreversibilities in opposing directions ($M-$). Predictions that are correct according to EcoCyc [11] are highlighted in green. Light green indicates predictions that we believe are correct but could not be confirmed. We did not seek validation for transporters (gray) as their exact stoichiometry is often unclear. Moreover, we ignored reactions already validated against 13C data and reactions that are potentially involved in substrate channeling (in that case, the direction of the reaction does not reflect the direction of the net flux).

Reaction	Condition	US	TFS	Explanation	Reference
ACCOAL	fru	FW	BW	Not found in ecocyc, DG° favors BW	
ACCOAL	glyc	FW	BW	Not found in ecocyc, DG° favors BW	
ACCOAL	pyr	FW	BW	Not found in ecocyc, DG° favors BW	
ACCOAL	succ	FW	BW	Not found in ecocyc, DG° favors BW	
ACt4pp	fru	FW	BW		
ACt4pp	glyc	FW	BW		
ACt4pp	pyr	FW	BW		
ACt4pp	succ	FW	BW		
CBMKr	fru	FW	BW	BW	https://ecocyc.org/ECOLI/NEW-IMAGE?type=PATHWAY&object=PWY0-41
CBMKr	glyc	FW	BW	BW	https://ecocyc.org/ECOLI/NEW-IMAGE?type=PATHWAY&object=PWY0-41
CBMKr	pyr	FW	BW	BW	https://ecocyc.org/ECOLI/NEW-IMAGE?type=PATHWAY&object=PWY0-41
CBMKr	succ	FW	BW	BW	https://ecocyc.org/ECOLI/NEW-IMAGE?type=PATHWAY&object=PWY0-41
CRNDt2rpp	glyc	FW	BW		
G3PD2	glyc	FW	BW	BW	https://ecocyc.org/ECOLI/NEW-IMAGE?type=REACTION&object=GLYC3PDEHYDROGBIOSYN-RXN
GLYAT	fru	FW	BW	BW	https://ecocyc.org/ECOLI/NEW-IMAGE?type=PATHWAY&object=THREONINE-DEG2-PWY
GLYAT	glyc	FW	BW	BW	https://ecocyc.org/ECOLI/NEW-IMAGE?type=PATHWAY&object=THREONINE-DEG2-PWY
GLYCLTt4pp	fru	FW	BW		
GLYCLTt4pp	glyc	FW	BW		
GLYCLTt4pp	pyr	FW	BW		
GLYCLTt4pp	succ	FW	BW		
NADH17pp	fru	FW	BW	Possibly BW (see thermodynamic assessment)	
NADH17pp	glyc	FW	BW	Possibly BW (see thermodynamic assessment)	
NADH17pp	pyr	FW	BW	Possibly BW (see thermodynamic assessment)	
NADH17pp	succ	FW	BW	Possibly BW (see thermodynamic assessment)	
PPM	fru	BW	FW	FW	https://ecocyc.org/ECOLI/NEW-IMAGE?type=PATHWAY&object=PWY0-1295
PPM	glyc	BW	FW	FW	https://ecocyc.org/ECOLI/NEW-IMAGE?type=PATHWAY&object=PWY0-1295
PPM	pyr	BW	FW	FW	https://ecocyc.org/ECOLI/NEW-IMAGE?type=PATHWAY&object=PWY0-1295
PPM	succ	BW	FW	FW	https://ecocyc.org/ECOLI/NEW-IMAGE?type=PATHWAY&object=PWY0-1295
PROt4pp	fru	FW	BW		
PROt4pp	glyc	FW	BW		
PROt4pp	pyr	FW	BW		
PROt4pp	succ	FW	BW		
PRPPS	fru	BW	FW	FW	https://ecocyc.org/ECOLI/NEW-IMAGE?type=REACTION&object=PRPPSYN-RXN
PRPPS	glyc	BW	FW	FW	https://ecocyc.org/ECOLI/NEW-IMAGE?type=REACTION&object=PRPPSYN-RXN
PRPPS	pyr	BW	FW	FW	https://ecocyc.org/ECOLI/NEW-IMAGE?type=REACTION&object=PRPPSYN-RXN
PRPPS	succ	BW	FW	FW	https://ecocyc.org/ECOLI/NEW-IMAGE?type=REACTION&object=PRPPSYN-RXN
TRSARr	fru	BW	FW	FW	https://ecocyc.org/ECOLI/NEW-IMAGE?type=REACTION&object=TSA-REDUCT-RXN
TRSARr	glyc	BW	FW	FW	https://ecocyc.org/ECOLI/NEW-IMAGE?type=REACTION&object=TSA-REDUCT-RXN
TRSARr	pyr	BW	FW	FW	https://ecocyc.org/ECOLI/NEW-IMAGE?type=REACTION&object=TSA-REDUCT-RXN
TRSARr	succ	BW	FW	FW	https://ecocyc.org/ECOLI/NEW-IMAGE?type=REACTION&object=TSA-REDUCT-RXN

Table SI 7: Evaluation of the predicted directions of reactions for which US and TFS predicted irreversibilities in opposing directions ($M+$). Predictions that are correct according to EcoCyc [11] are highlighted in green. Light green indicates predictions that we believe are correct but could not be confirmed. We did not seek validation for transporters (gray) as their exact stoichiometry is often unclear. Moreover, we ignored reactions already validated against 13C and reactions that are potentially involved in substrate channeling (in that case the direction of the reaction does not reflect the direction of the net flux).

Reaction	Condition	US	TFS	Explanation	Reference
ACCOAL	ac	FW	BW	Not found in ecocyc, DG° favors BW	
ACCOAL	fru	FW	BW	Not found in ecocyc, DG° favors BW	
ACCOAL	glc	FW	BW	Not found in ecocyc, DG° favors BW	
ACCOAL	glyc	FW	BW	Not found in ecocyc, DG° favors BW	
ACCOAL	pyr	FW	BW	Not found in ecocyc, DG° favors BW	
ACCOAL	succ	FW	BW	Not found in ecocyc, DG° favors BW	
ACKr	glyc	FW	BW	BW if not growing on acetate.	https://ecocyc.org/ECOLI/NEW-IMAGE?type=PATHWAY&object=PWY0-1312
ACt4pp	fru	FW	BW		
ACt4pp	glc	FW	BW		
ACt4pp	glyc	FW	BW		
ACt4pp	pyr	FW	BW		
ACt4pp	succ	FW	BW		
CBMKr	ac	FW	BW	BW	https://ecocyc.org/ECOLI/NEW-IMAGE?type=PATHWAY&object=PWY0-41
CBMKr	fru	FW	BW	BW	https://ecocyc.org/ECOLI/NEW-IMAGE?type=PATHWAY&object=PWY0-41
CBMKr	glc	FW	BW	BW	https://ecocyc.org/ECOLI/NEW-IMAGE?type=PATHWAY&object=PWY0-41
CBMKr	glyc	FW	BW	BW	https://ecocyc.org/ECOLI/NEW-IMAGE?type=PATHWAY&object=PWY0-41
CBMKr	pyr	FW	BW	BW	https://ecocyc.org/ECOLI/NEW-IMAGE?type=PATHWAY&object=PWY0-41
CBMKr	succ	FW	BW	BW	https://ecocyc.org/ECOLI/NEW-IMAGE?type=PATHWAY&object=PWY0-41
CRNDt2rpp	ac	FW	BW		
CRNDt2rpp	succ	FW	BW		
F6PA	ac	BW	FW	Not specified in ecocyc pathways	
F6PA	glyc	BW	FW	Not specified in ecocyc pathways	
F6PA	pyr	BW	FW	Not specified in ecocyc pathways	
F6PA	succ	BW	FW	Not specified in ecocyc pathways	
GLYAT	fru	FW	BW	BW	https://ecocyc.org/ECOLI/NEW-IMAGE?type=PATHWAY&object=THREONINE-DEG2-PWY
GLYAT	glc	FW	BW	BW	https://ecocyc.org/ECOLI/NEW-IMAGE?type=PATHWAY&object=THREONINE-DEG2-PWY
GLYAT	glyc	FW	BW	BW	https://ecocyc.org/ECOLI/NEW-IMAGE?type=PATHWAY&object=THREONINE-DEG2-PWY
GLYCLTt4pp	ac	FW	BW		
GLYCLTt4pp	fru	FW	BW		
GLYCLTt4pp	glc	FW	BW		
GLYCLTt4pp	glyc	FW	BW		
GLYCLTt4pp	pyr	FW	BW		
GLYCLTt4pp	succ	FW	BW		
NADH17pp	ac	FW	BW	Possibly BW (see thermodynamic assessment)	
NADH17pp	fru	FW	BW	Possibly BW (see thermodynamic assessment)	
NADH17pp	pyr	FW	BW	Possibly BW (see thermodynamic assessment)	
NADH18pp	ac	FW	BW	Possibly BW (see thermodynamic assessment)	
NADH18pp	fru	FW	BW	Possibly BW (see thermodynamic assessment)	
NDPK1	ac	BW	FW	FW	https://ecocyc.org/ECOLI/NEW-IMAGE?type=REACTION&object=GDPKIN-RXN
NDPK1	fru	BW	FW	FW	https://ecocyc.org/ECOLI/NEW-IMAGE?type=REACTION&object=GDPKIN-RXN
NDPK1	glyc	BW	FW	FW	https://ecocyc.org/ECOLI/NEW-IMAGE?type=REACTION&object=GDPKIN-RXN
POR5	ac	BW	FW	FW, distributed bottleneck with RNTR1*2	
PPM	fru	BW	FW	FW	https://ecocyc.org/ECOLI/NEW-IMAGE?type=PATHWAY&object=PWY0-1295
PPM	glc	BW	FW	FW	https://ecocyc.org/ECOLI/NEW-IMAGE?type=PATHWAY&object=PWY0-1295
PPM	succ	BW	FW	FW	https://ecocyc.org/ECOLI/NEW-IMAGE?type=PATHWAY&object=PWY0-1295
PROt4pp	ac	FW	BW		
PROt4pp	fru	FW	BW		
PROt4pp	glc	FW	BW		
PROt4pp	glyc	FW	BW		
PROt4pp	pyr	FW	BW		
PROt4pp	succ	FW	BW		
PRPPS	fru	BW	FW	FW	https://ecocyc.org/ECOLI/NEW-IMAGE?type=REACTION&object=PRPPSYN-RXN
PTAr	glyc	BW	FW	FW if not growing on acetate.	https://ecocyc.org/ECOLI/NEW-IMAGE?type=PATHWAY&object=PWY0-1312
TRSARr	ac	BW	FW	FW	https://ecocyc.org/ECOLI/NEW-IMAGE?type=REACTION&object=TSA-REDUCT-RXN
TRSARr	fru	BW	FW	FW	https://ecocyc.org/ECOLI/NEW-IMAGE?type=REACTION&object=TSA-REDUCT-RXN
TRSARr	glc	BW	FW	FW	https://ecocyc.org/ECOLI/NEW-IMAGE?type=REACTION&object=TSA-REDUCT-RXN
TRSARr	glyc	BW	FW	FW	https://ecocyc.org/ECOLI/NEW-IMAGE?type=REACTION&object=TSA-REDUCT-RXN
TRSARr	pyr	BW	FW	FW	https://ecocyc.org/ECOLI/NEW-IMAGE?type=REACTION&object=TSA-REDUCT-RXN
TRSARr	succ	BW	FW	FW	https://ecocyc.org/ECOLI/NEW-IMAGE?type=REACTION&object=TSA-REDUCT-RXN
VALTA	ac	FW	BW	BW, main path for valine synthesis	https://ecocyc.org/ECOLI/NEW-IMAGE?type=PATHWAY&object=VALSYN-PWY
VALTA	fru	FW	BW	BW, main path for valine synthesis	https://ecocyc.org/ECOLI/NEW-IMAGE?type=PATHWAY&object=VALSYN-PWY
VALTA	glc	FW	BW	BW, main path for valine synthesis	https://ecocyc.org/ECOLI/NEW-IMAGE?type=PATHWAY&object=VALSYN-PWY

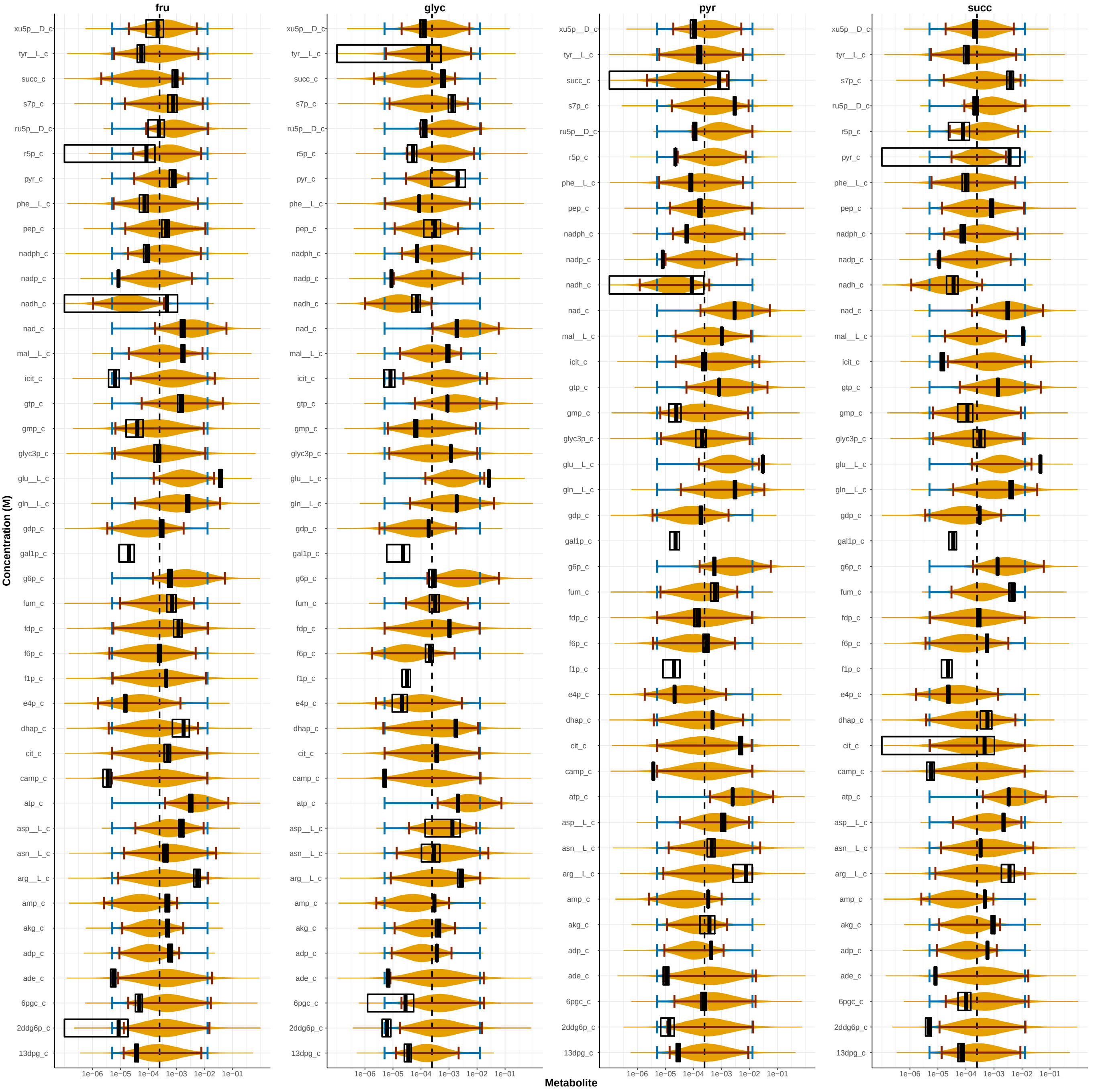

Figure SI 7: Distributions (orange) and 95% confidence intervals (red) of TFS predictions, predicted TMFA ranges (blue) and mean and 95% confidence intervals of the metabolomics data (black) are shown for all metabolites measured in [7]. Some concentrations could not be predicted because all the related reactions were blocked.

References

[1] A. Abadjieva, K. Pauwels, P. Hilven, and M. Crabeel. A new yeast metabolon involving at least the two first enzymes of arginine biosynthesis acetylglutamate synthase activity requires complex formation with acetylglutamate kinase. *Journal of Biological chemistry*, 276(46):42869–42880, 2001.

[2] C. J. Bélisle, H. E. Romeijn, and R. L. Smith. Hit-and-run algorithms for generating multivariate distributions. *Mathematics of Operations Research*, 18(2):255–266, 1993.

[3] B. Bulutoglu, K. E. Garcia, F. Wu, S. D. Minteer, and S. Banta. Direct evidence for metabolon formation and substrate channeling in recombinant tca cycle enzymes. *ACS chemical biology*, 11(10):2847–2853, 2016.

[4] B. Du, D. C. Zielinski, and B. O. Palsson. Estimating metabolic equilibrium constants: Progress and future challenges. *Trends in biochemical sciences*, 2018.

[5] P. Erdrich, R. Steuer, and S. Klamt. An algorithm for the reduction of genome-scale metabolic network models to meaningful core models. *BMC systems biology*, 9(1):48, 2015.

[6] A. Gelman, J. B. Carlin, H. S. Stern, D. B. Dunson, A. Vehtari, and D. B. Rubin. *Bayesian data analysis*. CRC press, 2013.

[7] L. Gerosa, B. R. H. van Rijsewijk, D. Christodoulou, K. Kochanowski, T. S. Schmidt, E. Noor, and U. Sauer. Pseudo-transition analysis identifies the key regulators of dynamic metabolic adaptations from steady-state data. *Cell systems*, 1(4):270–282, 2015.

[8] W. G. Hearl and J. E. Churchich. Interactions between 4-aminobutyrate aminotransferase and succinic semialdehyde dehydrogenase, two mitochondrial enzymes. *The Journal of biological chemistry*, 259 18:11459–63, 1984.

[9] C. S. Henry, L. J. Broadbelt, and V. Hatzimanikatis. Thermodynamics-based metabolic flux analysis. *Biophysical journal*, 92(5):1792–1805, 2007.

[10] C. L. James and R. E. Viola. Production and characterization of bifunctional enzymes. substrate channeling in the aspartate pathway. *Biochemistry*, 41(11):3726–3731, 2002.

[11] I. M. Keseler, A. Mackie, A. Santos-Zavaleta, R. Billington, C. Bonavides-Martínez, R. Caspi, C. Fulcher, S. Gama-Castro, A. Kothari, M. Krummenacker, et al. The EcoCyc database: reflecting new knowledge about escherichia coli k-12. *Nucleic acids research*, 45(D1):D543–D550, 2017.

[12] G. J. Kortstee, K. J. Appeldoorn, C. F. Bonting, E. W. van Niel, and H. J. Van Veen. Ecological aspects of biological phosphorus removal in activated sludge systems. In *Advances in Microbial Ecology*, pages 169–200. Springer, 2000.

[13] H. Lauble and C. D. Stout. Steric and conformational features of the aconitase mechanism. *Proteins: Structure, Function, and Bioinformatics*, 22(1):1–11, 1995.

[14] C. Marco-Marín, F. Gil-Ortiz, I. Pérez-Arellano, J. Cervera, I. Fita, and V. Rubio. A novel two-domain architecture within the amino acid kinase enzyme family revealed by the crystal structure of escherichia coli glutamate 5-kinase. *Journal of molecular biology*, 367(5):1431–1446, 2007.

[15] I. I. Mathews, T. J. Kappock, J. Stubbe, and S. E. Ealick. Crystal structure of escherichia coli pure, an unusual mutase in the purine biosynthetic pathway. *Structure*, 7(11):1395–1406, 1999.

[16] P. Salvy, G. Fengos, M. Ataman, T. Pathier, K. C. Soh, and V. Hatzimanikatis. pyTFA and matTFA: a python package and a matlab toolbox for thermodynamics-based flux analysis. *Bioinformatics*, 35(1):167–169, 2019.

[17] D. Szklarczyk, A. L. Gable, D. Lyon, A. Junge, S. Wyder, J. Huerta-Cepas, M. Simonovic, N. T. Doncheva, J. H. Morris, P. Bork, et al. STRING v11: protein–protein association networks with increased coverage, supporting functional discovery in genome-wide experimental datasets. *Nucleic acids research*, 47(D1):D607–D613, 2018.

[18] A. D. Vinogradov. Catalytic properties of the mitochondrial NADH–ubiquinone oxidoreductase (complex I) and the pseudo-reversible active/inactive enzyme transition. *Biochimica et Biophysica Acta (BBA)-Bioenergetics*, 1364(2):169–185, 1998.

[19] V. Vojinović and U. von Stockar. Influence of uncertainties in pH, pMg, activity coefficients, metabolite concentrations, and other factors on the analysis of the thermodynamic feasibility of metabolic pathways. *Biotechnology and bioengineering*, 103(4):780–795, 2009.

[20] H. P. Williams. *Model building in mathematical programming*. John Wiley & Sons, 2013.

[21] F. Wu and S. Minteer. Krebs cycle metabolon: structural evidence of substrate channeling revealed by cross-linking and mass spectrometry. *Angewandte Chemie International Edition*, 54(6):1851–1854, 2015.

[22] W. Zhang, M. Zhang, C. Gao, Y. Zhang, Y. Ge, S. Guo, X. Guo, Z. Zhou, Q. Liu, Y. Zhang, et al. Coupling between d-3-phosphoglycerate dehydrogenase and d-2-hydroxyglutarate dehydrogenase drives bacterial l-serine synthesis. *Proceedings of the National Academy of Sciences*, 114(36):E7574–E7582, 2017.
